# Microarray based gene expression analysis of *Sus Scrofa* duodenum exposed to zearalenone: significance to human health

**DOI:** 10.1186/s12864-016-2984-8

**Published:** 2016-08-17

**Authors:** Cornelia Braicu, Roxana Cojocneanu-Petric, Ancuta Jurj, Diana Gulei, Ionelia Taranu, Alexandru Mihail Gras, Daniela Eliza Marin, Ioana Berindan-Neagoe

**Affiliations:** 1 Research Center for Functional Genomics, Biomedicine and Translational Medicine, “Iuliu Hatieganu”, University of Medicine and Pharmacy, Marinescu 23 Street, No. 23, 400012 Cluj-Napoca, Romania; 2 Department of Functional Genomics and Experimental Pathology, The Oncological Institute “Prof. Dr. Ion Chiricuta”, Republicii Street, No. 34-36, 401015 Cluj-Napoca, Romania; 3 Laboratory of Animal Biology, National Institute for Research and Development for Biology and Animal Nutrition, Calea Bucuresti No. 1, Balotesti, Ilfov 077015 Romania; 4 MEDFUTURE -Research Center for Advanced Medicine, University of Medicine and Farmacy Iuliu-Hatieganu, Cluj-Napoca, Romania

**Keywords:** Zearalenone, Duodenum, *Sus scrofa*, Extrapolation to human, Health, Microarray

## Abstract

**Background:**

Zearalenone (ZEA) is a secondary metabolite produced by Fusarium species. ZEA was proved to exert a wide range of unwanted side effects, but its mechanism of action, particularly at duodenum levels, remains unclear. In our study based on the microarray technology we assessed the alteration of gene expression pattern *Sus scrofa* duodenum which has been previously exposed to ZEA. Gene expression data was validated by qRT-PCR and ELISA. The gene expression data were further extrapolated the results to their human orthologues and analyzed the data in the context of human health using IPA (Ingenuity Pathways Analysis).

**Results:**

Using Agilent microarray technology, we found that gene expression pattern was significantly affected by ZEA exposure, considering a 2-fold expression difference as a cut-off level and a *p*-value < 0.05. In total, we found 1576 upregulated and 2446 downregulated transcripts. About 1084 genes (764 downregulated and 751 overexpressed) were extrapolated to their human orthologues. IPA analysis showed various altered key cellular and molecular pathways. As expected, we observed a significant alteration of immune response related genes, MAPK (mitogen activate protein kinases) pathways or Toll-Like Receptors (TLRs). What captured our attention was the modulation of pathways related to the activation of early carcinogenesis.

**Conclusions:**

Our data demonstrate that ZEA has a complex effect at duodenum level. ZEA is able to activate not only the immune response related genes, but also those relate to colorectal carcinogenesis. The effects can be more dramatic when connected with the exposure to other environmental toxic agents or co-occurrence with different microorganisms.

**Electronic supplementary material:**

The online version of this article (doi:10.1186/s12864-016-2984-8) contains supplementary material, which is available to authorized users.

## Background

Zearalenone (ZEA) is a secondary metabolite produced by certain species that belong to the genus *Fusarium*, a common group of fungus species [1, 2]. These fungi are common contaminants present is almost all types of crops, including those used as feed for farm animal diets as well as cereals consumed by humans [3–5]. The ZEA mycotoxin has a particular way of exerting its effects by interfering in the physiological estrogen signaling pathways, since its chemical structure is similar to that of the estrogenic hormone 17-β-estradiol [6–8]. Among the different farm animals, pigs proved to be highly sensitive to the effects of ZEA mycotoxin via contaminated feed. They are known to display various deleterious effects caused by this mycotoxin, but, regardless of the estrogenic activity of ZEA, many of the observed damages are, presumably, not always mediated by the estrogen receptor [9, 10]. Also, the histology, anatomy, physiology – and consequently the pathology – of this species are very similar to that of humans, making it a suitable model for the study of various diseases and physio-pathological processes [11, 12].

The intestine is the interface between the organism and the whole exposome, so it interacts with the existent microflora and with the pathogenic agents that may occur [13–15]. These particular tissues were chosen because, as part of the gastro-intestinal (GI) tract, they are involved in the immune response and inflammation on the one hand, and they are among the most exposed to the action of the contaminants on the other hand. This makes these organs suitable subjects for this microarray profiling study [16–20].

The animal model used for this study is the pig, due to the previously mentioned genetic similarity between *Sus scrofa* and *Homo sapiens*. At the same time, pigs consume high quantities of maize, a cereal which is very prone to *Fusarium* mycotoxin contamination [21], and which is also a raw material in the diet of humans. Such studies are of particular interest because humans also consume high quantities of different cereals which are, many times, contaminated with various mycotoxins such as ZEA [9, 22]. Even though there are regulations regarding the maximum tolerated values of food contaminating agents, including ZEA [23], some such toxins have shown increased resistance to decontaminating procedures, and a wide range of side effects [24, 25]. Therefore it is important to acquire a better understanding of the influence that ZEA has on the health of humans and their surrounding environment.

## Results

### Evaluation of duodenal gene expression pattern

A significant supposition in many toxicological investigations is that the molecular states of organisms indicate their biological responses to a particular toxin, like in our case ZEA mycotoxin. Using the Agilent microarray technology, we found that gene expression was considerably affected by ZEA at duodenum level, considering 2-fold expression difference as a cut-off level and *p*-value < 0.05. In total, we found 4023 transcripts with an altered expression level, from which 1576 were upregulated and 2446 downregulated. *Sus scrofa* is a key mammalian model system for studying complex human diseases and therefore it is useful to assess the impact of the toxin on this model, then to extrapolate the gene expression profile and to analyze the data in the context of human health. We were able to obtain the human orthologues for about 1084 genes (764 downregulated and 751 overexpressed).

### Microarray data validation by qRT-PCR

**Fig. 1:**
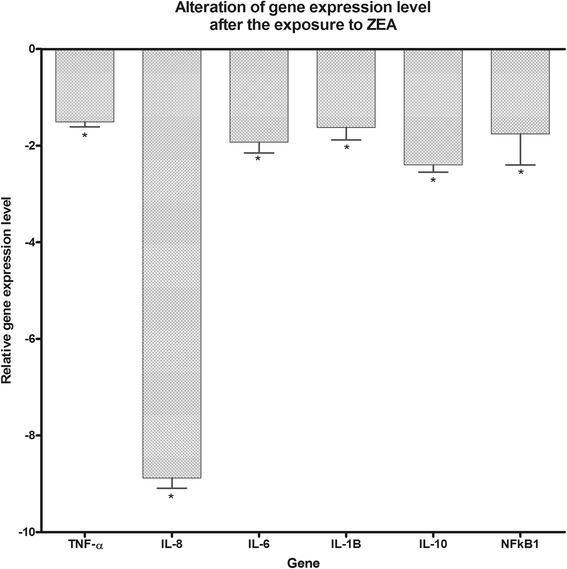
qRT-PCR data validation of the microarray data

The obtained microarray data were validated by qRT-PCR. Therefore four genes were selected (NFKB1, IL-6, TNF-α and SOD2) and three housekeeping genes (ACTB, GAPDH, B2M) were used for the normalization of the data. qRT-PCR data confirm the microarray downregulation expression of these genes, moreover it shows a good correlation among them (Fig. 1).

### The impact of ZEA exposure on cytokine protein expression at duodenum levels

**Fig. 2:**
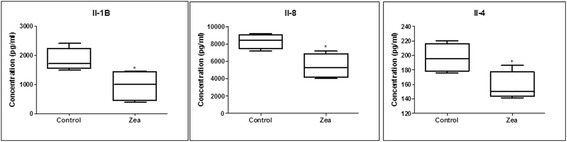
Protein expression quantification by ELISA at duodenum level for IL-1B, IL-8 and IL-4 for the control group and the group exposed to ZEA

The microarray data displayed previously showed an alteration of molecules involved in immune response. Therefore, we assessed the protein level for IL-1β, IL-8, IL-4 and TNF-α for cellular lysates. As it can be seen from Fig. 2, we observed a downregulation of IL-1β, IL-8, IL-4 at protein level as a response to ZEA exposure. The level of TNF-α was under the limit of detection provided by our method.

### Network analysis

A primary goal of the study was to identify the possible implications of the altered genes at cellular and molecular level, as well as the related functions and pathways that might be mediated by ZEA (Table 1). To accomplish this objective in an unbiased manner, we performed IPA analysis for all the genes with and altered expression levels that were extrapolated to their human orthologues. This facilitated the assessment of the potential impact by analyzing the networks, biological functions, and canonical pathways. Additional file 1: Figure S1 presents the top canonical pathways, based on the overlap value, and displays the genes related to the alteration of Toll-Like Receptors (TLRs) and the activation of the inflammatory cytokine in parallel with the alteration of the expression level for the adhesion molecules. In Additional file 2: Figure S2 are emphasized the altered genes related to MAPK (mitogen activate protein kinases), an early event of carcinogenesis, fact demonstrated in Additional file 3: Figure S3. Also, we observed alterations in GAP junction signaling (Additional file 4: Figure S4).

**Table 1:** Top 4 canonical pathway targeted by ZEA at duodenum level

	Canonical pathway	*p*-value	Overlap
1	TREM1 Signaling	1.45E-06	26.7 % 20/75
	Dopamine-DARPP32 Feedback in cAMP Signaling	5.22E-06	19.3 % 31/161
2	Cancer Regulation by Stathmin1	4.29E-06	18.3 % 35/191
3	Role of Macrophages, Fibroblasts and Endothelial Cells in Rheumatoid Arthritis	1.81E-08	18.1 % 54/298
4	Protein Kinase A Signaling	1.63E-06	15.3 % 59/385

**Fig. 3:**
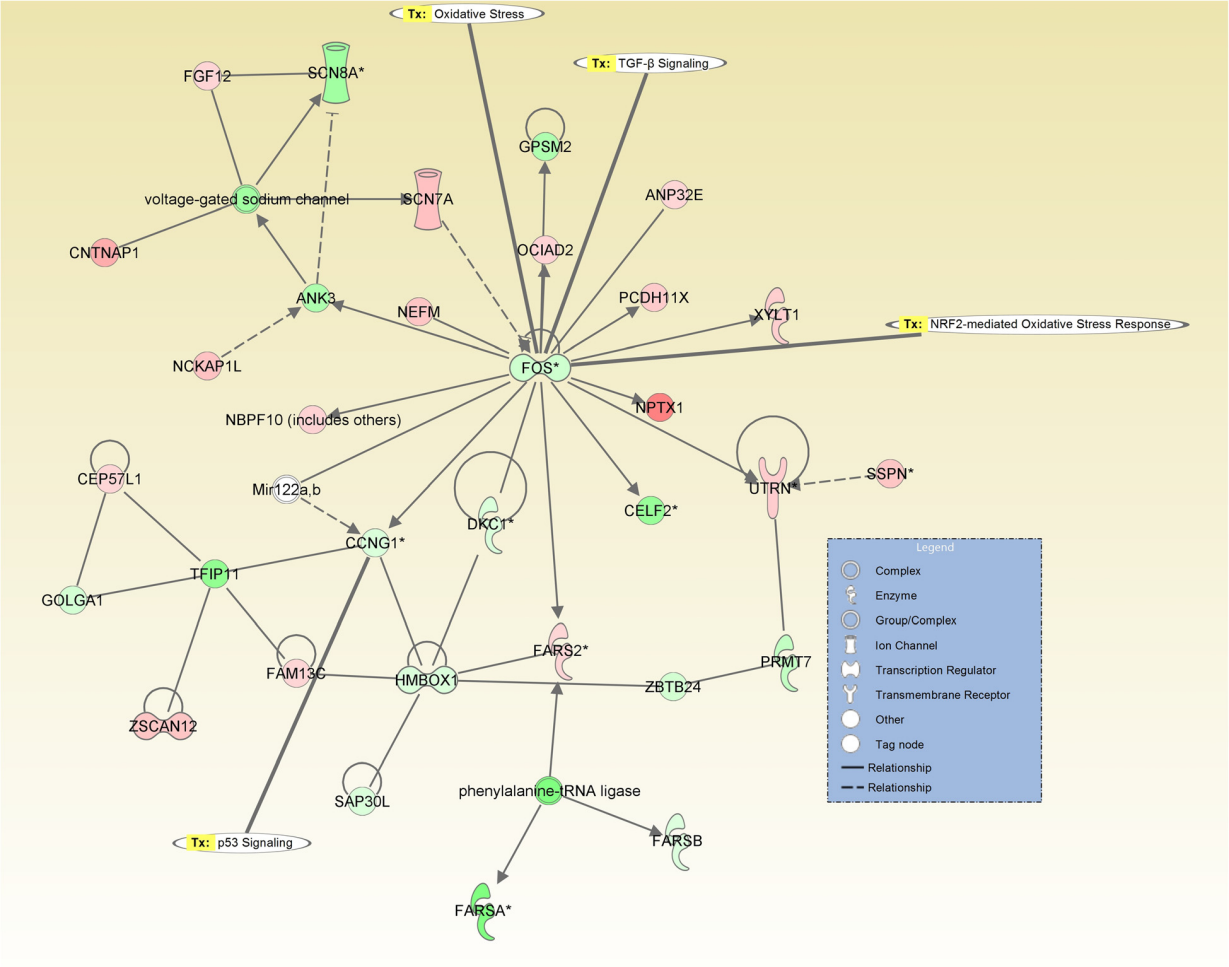
Gene network interaction generated using IPA, as response to ZEA exposure at duodenul level, as key central molecule is FOS, being relate to oxidative stress response and TGFB1 pathways. The altered expressed genes are displayed in red and green, based on fold change expression level, meanwhile the gene in grey are retrieved in literature to interact

**Fig. 4:**
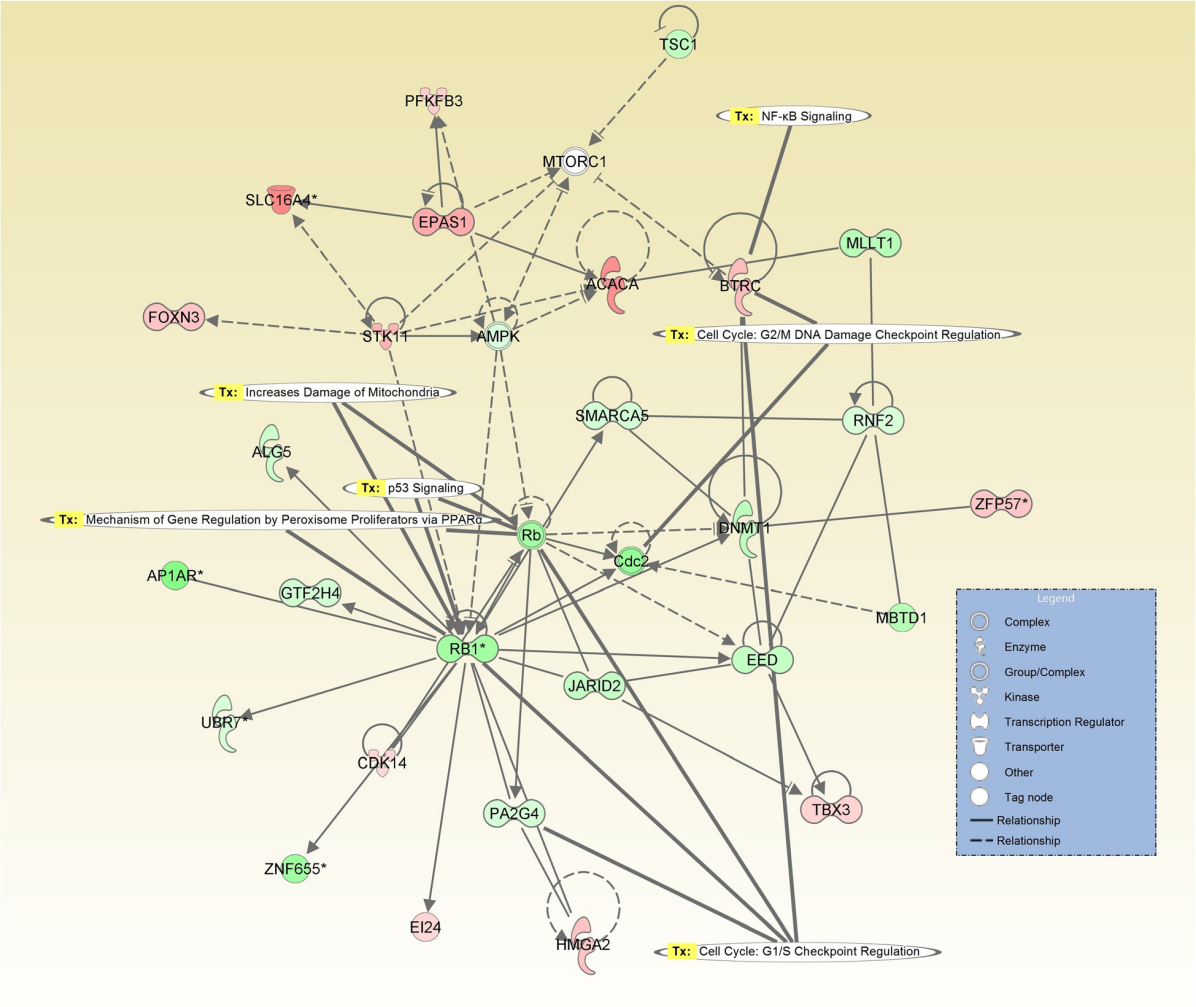
Gene network interaction generated using IPA, as response to ZEA exposure at duodenum level, as key central molecule is RB, being relate to alteration of the cell cycle or NFkB pathways. The altered expressed genes are displayed in red and green, based on fold change expression level, meanwhile the gene in grey are retrieved in literature to interact

The analysis of probe sets that are differentially expressed with a fold change greater than 2 revealed 24 altered pathways, specific for the toxin exposure. Most of the altered networks were related to the alteration of normal cell status, the most significant network containing 34 focus molecules (Fig. 3), with the core of this network being the FOS gene. Another interesting network is that represented by the one with the ID7 (Table 2), related to carcinogenesis, cell death and organism damage (Figs. 3 and 4 ), the core or this network being represented by ER. A curious aspect of the gene expression alteration at duodenum level is represented by the network with the ID20 (Table 1), where the core of the network is the RB1 gene; this gene is recognized to be involved in TGFB1 or p53 signaling cell cycle regulation, but also to be related to mitochondrial damage.

**Table 2:** Top disease and function altered as response to ZEA exposure

ID	Top diseases and functions	Score	Focus molecules
1	Cell Morphology, Cellular Assembly and Organization, Cellular Function and Maintenance	40	34
2	Metabolic Disease, Neurological Disease, Organismal Injury and Abnormalities	38	33
3	Auditory Disease, Hereditary Disorder, Neurological Disease	38	33
4	Molecular Transport, Hereditary Disorder, Connective Tissue Disorders	35	32
5	Digestive System Development and Function, Endocrine System Development and Function, Organ Morphology	31	30
6	Cellular Movement, Cell-To-Cell Signalling and Interaction, Nervous System Development and Function	31	30
7	Cancer, Cell Death and Survival, Organismal Injury and Abnormalities	31	30
8	Gene Expression, Protein Synthesis, RNA Post-Transcriptional Modification	29	29
9	Cell Death and Survival, Cell Morphology, Infectious Disease	29	29
10	Cardiovascular System Development and Function, Cancer, Cardiovascular Disease	29	29
11	Connective Tissue Disorders, Immunological Disease, Inflammatory Disease	28	28
12	Tissue Development, Post-Translational Modification, Cell Cycle	28	28
13	Cell Cycle, DNA Replication, Recombination, and Repair, Cellular Compromise	26	27
14	Nucleic Acid Metabolism, Small Molecule Biochemistry, Cell Cycle	26	27
15	DNA Replication, Recombination, and Repair, Cell Cycle, Reproductive System Development and Function	26	27
16	Cellular Growth and Proliferation, Embryonic Development, Organ Development	26	27
17	RNA Post-Transcriptional Modification, Developmental Disorder, Hereditary Disorder	26	27
18	Developmental Disorder, Hereditary Disorder, Skeletal and Muscular Disorders	26	27
19	Protein Synthesis, Cancer, Organismal Injury and Abnormalities	26	27
20	Energy Production, Lipid Metabolism, Small Molecule Biochemistry	26	27
21	Cellular Assembly and Organization, Inflammatory Disease, Inflammatory Response	24	26
22	Lipid Metabolism, Molecular Transport, Small Molecule Biochemistry	24	26
23	RNA Post-Transcriptional Modification, Carbohydrate Metabolism, Cellular Compromise	24	26
24	Amino Acid Metabolism, Small Molecule Biochemistry, Cell Signaling	24	26
25	Infectious Disease, Organismal Injury and Abnormalities, Dermatological Diseases and Conditions	24	26

## Discussion

ZEA mycotoxin is absorbed up to 90 % in the upper part of the GIT (gastrointestinal tract), and it goes into enterohepatic circulation as other mycotoxins [26]. One of the important roles of the GIT is its function as an immune barrier. This function is accomplished through a number of particularities. Firstly, it possesses its own immune system and it is estimated that up to 70 % of the immune defenses of the organism are located in the intestine. Secondly, its morphology plays a role as a physical barrier through tight junctions (TJs) formed mainly from occludin and claudin isoforms proteins, and gap junctions (GJs) that permit the transfer of ions, nucleotides and other small molecules between adjacent cells, and are formed mainly by connexins. Last but not least, the intestinal microbiota plays a very important role in protecting against pathogen invasion [27]. Moreover, the physical barrier can be disrupted due to defective TJs and GJs. ZEA has been shown to reduce mRNA levels of occludin and claudin-4 and also the protein levels of connexin [28], a mechanism that is altered by cytotoxic and carcinogenic agents [29], fact demonstrated also by present study, via multiple mechanisms relate to cell-to-cell communication.

By quantifying the modified expression of tens of thousands of genes, we can recognize mechanistically-relevant genes, leading to a better understanding of the ZEA toxicological effects at duodenum level.

ZEA was proved to affect the alteration of several genes involves in inflammatory response, oxidative stress, proteinases or other relevant genes [30]. The capacity to activate oxidative stress was proved also in an in vitro study [31]. It was reported that ZEA has pro-apoptotic effects, by increasing the Bax/Bcl-2 ration [32]. ZEN was observed to be involved in hepatic injury via Nrf2/ARE signaling pathway [33], mechanism altered also at intestinal level. Therefore, continuous exposure might be related to the activation of the tumor-promoting inflammatory mechanisms [30], expressed by the inflammatory cytokines or by the antioxidant enzyme, these being validated by qRT-PCR. It is clear that ZEA leads to an inflammatory microenvironment, and this can be correlated with increased mutation rates, all these representing early carcinogenic events. This fact sustained by other environmental toxic agents can be an essential in the activation of tumor initiation [34]. In the case of HCT116 cells it was observed that even at low concentration ZEA increase cell proliferation, colony formation and cell migration [35].

ZEA compounds were observed to exert estrogenic effects [7, 36], even at nM concentration [36], fact confirmed by the present study. The estrogenic effects seem to be altered via Erk1/2 phosphorylation, mechanism, in parallel with the sustaining of cell proliferation [36]. ZEN could promote the autocrine action or expression of the ghrelin gene in piglet ovary [37].

Other studies confirmed the activation of MAPK and oxidative stress related genes as response to ZEA in MCF-7 breast cancer cells, [38] and macrophages [31], this findings being confirmed by this present in vivo study at duodenum level. The oxidative species seems to be generated via p53, JNK or p38 pathways [31, 39]. In our study NFKB1 was found to be down-regulated, also it was connected with a proapoptotic effect in colon carcinoma cells. c-Myc is an early response gene with important regulatory functions in the cell cycle, and known to be regulated in colon cancer. c-Myc was downregulated by ZEA exposure. In the same time, the IPA network analysis showed the complex action of ZEA, the analysis being more focused on the alteration of the mitochondrial pathways connected with endocrine dysfunction [40]. There are several classes of altered genes, such as Ras-related genes, kinases/phosphatases and their binding proteins, genes retrieved also in study. These Ras pathways contribute to cell growth and proliferation [36].

Alteration of Stathmin1 pathways are connected with invasion and migration capacity in digestive cancers [41]. The oncogenic role of the Stathmin1 pathways was proved to be related via miR-223 [42]. In a recent review paper Reuter et al., (2010) present a clear connection between oxidative stress and the activation of the transcription factors (NFkB, AP-1, HIF-1α, PPAR-γ, Wnt and Nrf2) [43]. The present study confirms that the activation of these transcription factors is related to the alteration of other multiple genes like growth factors, cytokines/chemokines, cell cycle related genes, cell proliferation or differentiation anti-inflammatory molecules, or those in invasion and migration. From the top 10 genes that were highly expressed in the case of duodenum exposure to ZEA, a representative one is CTNNA1. CTNNA1, along with other MAPK related genes [31, 39], have been proved to be involved in gastric cancer [44]. NFAT-signaling pathway activation was proved to be implicated in aggressive forms of inflammatory pathologies. Moreover, this pathway was proved to have a significant role in inflammatory breast cancer [45].

The results remains to be further investigated particular on ZEA derivatives, a recent study present antitumoral effects of the semisynthetic zearalenone analogues [46]. Therefore the mechanisms of transformation and detoxification in parallel with the combinatorial effect need to be taken in account [47]. The procarcinogenic effect of ZEA was also studied by Ouanes et al., 2003 [48], and should take into account the accumulation in organism and the time required for metabolisation of this toxins [49]. Novel monitoring method based on urinary excretion of ZEA metabolites should be developed [50].

Policies and regulations of the mycotoxin need to be assessed in the context of the exposome exposure, not only guided by compound exposure and evaluation of the dose-response analyses in risk assessment [51, 52]. The impact of mycotoxins on human health needs to be considered in a transformative mode to take into account the whole exposome exposure [52]. Even in the case of a single toxic exposure (ZEA), the effect on gene expression is significant at duodenum level. There are several clues that environmental toxic exposure implies [51], including the case of ZEA. This toxin can interact and affect more dramatically health status that is presented by microarray data. ZEA is only one component of the exposome, therefore ZEA toxic effects can be more dramatic in the context of multiple exposures.

## Conclusion

As ZEA caused alteration of gene expression, chronic exposure can create a metabolic environment that allows mutated cells to acquire a selective advantage. Our data demonstrate that ZEA activates not only the immune related genes response, but also those related to carcinogenesis. The effect can be more dramatic when is connected with other environmental toxic agents and this should be taken into account for further implementation of novel norms for the control of this toxin.

## Methods

### Animals and treatments

Two groups of weaned TOPIG piglets [(Landrace × Large White) × (Duroc × Pietrain)], *n* = 6 per group/pen) with an initial average body weight of 9.88 ± 0.2 Kg and derived from the experimental farm of the National Research Institute for Biology and Animal Nutrition, Balotesti Romania were used in this study. The animals individually identified by ear tag were housed in pens and fed with experimental diets: a control diet without mycotoxin or a diet contaminated with ZEA (100 ppb) for 30 days. Assigned diet and water were provided *ad libitum* every day of the experiment. Pigs were observed twice daily and no clinical signs or death was recorded throughout the entire experimental period. At the end of the experiment, animals were stunned and slaughtered in an EU-licensed abattoir according with the EU Council Directive 2010/63/CE. Organ samples were collected on ice from all animals, weighed and were stored at –80 °C until the analyses.

Animals were raised in agreement with the Romanian Law 43/2014 for handling and protection of animals used for experimental purposes and the EU Council Directive 98/58/EC concerning the protection of farmed animals. The study protocol was accepted by the Ethical Committee of the National Research-Development Institute for Animal Nutrition and Biology, Balotesti, Romania (Ethical Committee no. 52/2014). At the end of the research period, animals were stunned and slaughtered in an EU-licensed abattoir in accordance with the EU Council Directive 2010/63/CE.

### RNA extraction and quality control

The duodenum tissue collected from weaned pigs exposed to ZEA for 33d was disrupted using a mechanical homogenizer, in the presence of TRI Reagent (Sigma-Aldrich), and the total RNA was extracted following the phase separation protocol with chloroform and isopropanol, according to the manufacturer’s protocol. The RNA thus obtained underwent a further purification step with the RNeasy Micro Kit from Qiagen, which uses silicagel spin columns to generate high quality RNA from small quantities of tissue. The precise concentrations and integrity of extracted nucleic acids, necessary for the subsequent microarray step were then evaluated using the NanoDrop2000 (Thermo Scientific) and the Bioanalyzer 2100 (Agilent Technologies).

### Microarray

The microarray probes were synthesized from equal quantities of 100 ng of total RNA, by using Agilent Low Input Quick Amp Labeling Kit (5190-2305) according to the manufacturer’s protocol. Subsequent to this step, the hybridization products were purified using RNeasy Mini kit (Qiagen). Probe quality control was executed using the NanoDrop2000 spectrophotometer (Thermo Scientific), all the samples having a specific activity higher than 6 pmol/μl Cy3/μg cRNA (specific activity > 8 pmol Cy3/μg cRNA). The fragmentation and hybridization were performed based on the Agilent one color protocol.

The samples were hybridized (17 h at 65 °C) on a custom microarray slide using SureDesign, containing up of 60mer oligos for over 59,000 probes representative for a high number of *Sus scrofa* transcripts (AMADID 056850). The microarray slides were scanned with the SureScan Microarray Scanner (1x60k array slides with 61 × 21 mm, resolution 3 μM) from Agilent and the images were processed with Feature Extraction 11.0.1.1 software. GeneSpring GX v.13.0 from Agilent was used for data analysis, which started with the removal of control probes and quantile normalization. A threshold of 2.0 was applied for the fold change, and then the moderated *t test* was used, together with the FDR (False Discovery Rate) correction method and extrapolated to human as described by Petric et al. [53].

The information related to homology annotation for all the transcripts sequence for S. scrofa and humans was used NCBI database (www.ncbi.nlm.nih.gov) and BLAST. The analysis in the context of human health for the altered genes as response to ZEA exposure was done by ingenuity pathway analysis (IPA; htp://www.ingenuity.com).

### qRT-PCR data validation

For the validation of the gene profiles provided by microarray analysis, the expression levels of five randomly selected genes: TNF-α (Tumor Necrosis Factor α), IL-8 (Interleukin 8), IL-6 (Interleukin 6), IL-1β (Interleukin 1 beta), IL-10 (Interleukin 10) and NFkB1 (nuclear factor of kappa light polypeptide gene enhancer in B-cells 1) were measured by real time RT-qPCR in all samples used for microarray analysis. 1 μg of total RNA extracted from each sample was used to generate cDNA using M-MLV Revers Trascriptase kit (Invitrogen, Life Technologies), according to the manufacturer’s recommendations. Quantitative fluorescent real-time PCR reactions were set up in a total volume of 20 μl using 10 μl of cDNA (diluted 1:10 with nuclease-free water), SYBR Green PCR Master Mix (Applied Biosystems, Life Technologies), 0.3 μM each of gene-specific primer and performed in the Rotor- Gene-Q (QIAGEN GmbH, Germany) machine. The primer pairs used in the present study, listed in Table 3, were obtained from Eurogentec (San Diego, USA). The cycling conditions were set up according to manufacturer’s recommendation, as follows: UDG pre-treatment at 50 °C for 2 min, initial denaturation step at 95 °C for 15 s, followed by 40 cycles of 95 °C for 15 s, 60 °C for 60s with a single fluorescence measurement. The specificity of the PCR products was confirmed by analysis of the dissociation curve. The melting curve programme consisted of temperatures between 60 and 95 °C with a heating rate of 0.1 °C/s and continuous fluorescence measurement. Negative controls were used for each primer pair. The relative quantification of gene expression changes were quantified using the comparative method. The expression levels of two reference genes, GAPDH and β-actin were used for data normalisation. These reference genes were experimentally validated for duodenum tissues and the lack of treatment effect and expression variation was the criteria for reference gene choice. The results were expressed as relative fold change (Fc) in comparison with control samples.

**Table 3:** qPCR primer sequences and characteristics

Gene	Accesion no.	Primer source	Primer sequence (5′ → 3′)	Orientation	Tm (°C)	Amplicon lenght (bp)	References
TNF-α	NM_214022	Pig	ACTGCACTTCGAGGTTATCGG	forward	60	118	[54]
			GGCGACGGGCTTATCTGA	reverse	60		
IL-8	NM_213867.1	Pig	GCTCTCTGTGAGGCTGCAGTTC	forward	58	79	[54]
			AAGGTGTGGAATGCGTATTTATGC	reverse	54		
IL-6	NM_214399	Pig	GGCAAAAGGGAAAGAATCCAG	forward	57	87	[54]
			CGTTCTGTGACTGCAGCTTATCC	reverse	61		
IL-1β	NM_214055	Pig	ATGCTGAAGGCTCTCCACCTC	forward	62	89	[55]
			TTGTTGCTATCATCTCCTTGCAC	reverse	59		
IL-10	NM_214041.1	Pig	GGCCCAGTGAAGAGTTTCTTTC	forward	54	51	[54]
			CAACAAGTCGCCCATCTGGT	reverse	55		
NFkB1	NM_001048232.1	Pig	TCGCTGCCAAAGAAGGACAT	forward	54	101	[55]
			AGCGTTCAGACCTTCACCGT				
GAPDH	NM_001206359.1	Pig	ACTCACTCTTCTACCTTTGATGCT	forward	49	100	[54]
			TGTTGCTGTAGCCAAATTCA	reverse	56		
B-actina	NM_213978.1	Pig	GGACTTCGAGCAGGAGATGG	forward	60	230	[56]
			GCACCGTGTTTGCGTAGAGG	reverse	62		

### Cytokine concentration evaluation (ELISA)

Organ lysate was prepared as described by Taranu et al., (2014) [10] and cytokine concentrations in the supernatant were determined by ELISA. Briefly, the purified fraction of anti-swine cytokines (IL-1β, IL-8, IL-4) were used as capture antibodies in conjunction with biotinylated anti-swine antibodies (R&D system). Streptavidin-HRP (Sigma) and TMB (Sigma) were used for detection. Absorbance was read at 450 nm using an ELISA plate reader (Tecan, Sunrise, Austria). Recombinant swine cytokine proteins were used as standard and results were expressed as picograms of cytokine/mL.

## Abbreviations

ACTB, Beta-actin; AP-1, Activator protein 1; ARE, antioxidant response element; B2M, Beta-2-Microglobulin; Bax, BCL2 Associated X Protein; Bcl-2, B-cell lymphoma 2; CTNNA1, Catenin Alpha 1; ER, Estrogen Recptor; Erk1/2, extracellular signal–regulated kinases 1/2; FDR, False Discovery Rate; GAPDH , Glyceraldehyde-3-Phosphate Dehydrogenase; GAPDH, Glyceraldehyde-3-Phosphate Dehydrogenase; GIT, gastrointestinal tract; GJs, gap junctions; HIF-1α , Hypoxia-inducible factor 1-alpha; IL-10, Interleukin 10; IL-1β, Interleukin 1 beta; IL-4, Interleukin 4; IL-6, Interleukin 6; IL-8, Interleukin 8; IPA, Ingenuity Pathways Analysis; JNK, Jun amino-terminal kinases; MAPK, mitogen activate protein kinases; NFAT, Nuclear factor of activated T-cells; NFKB1, nuclear factor of kappa light polypeptide gene enhancer in B-cells 1; Nrf2, nuclear erythroid 2-related factor 2; PPAR-γ, Peroxisome Proliferator-activated Receptor γ; RB1, retinoblastoma; SOD2, Superoxide dismutase 2; TGFB1, Transforming Growth Factor Beta 1; TJs, tight junctions; TLRs - *Toll-Like Receptors;* TNF-α, Tumor Necrosis Factor α; ZEA, Zearalenone

## Additional files

Additional file 1: Figure S1. Alteration of Toll-Like Receptors (TLRs) and the activation of the inflammatory cytokine in parallel with the alteration of the expression level for the adhesion molecules as response to ZEA exposure; pathway generated using IPA (Ingenuity Pathway Analysis). (JPG 5492 kb) Additional file 2: Figure S2. Alteration of genes related to MAPK (mitogen activate protein kinases), an early event of carcinogenesis, fact demonstrated as response to ZEA exposure; pathway generated using IPA. (JPG 2327 kb) Additional file 3: Figure S3. Activation of colorectal carcinogenic mechanism as response to ZEA exposure; pathway generated using IPA. (JPG 5802 kb) Additional file 4: Figure S4. Alterations in GAP junction signaling with signification on the increasing the susceptibility to bacterial infection; pathway generated using IPA. (JPG 2711 kb)
