# Supplementary figures and images for: Microarray based gene expression analysis of Sus Scrofa duodenum exposed to zearalenone: significance to human health
Source: BMC Genomics. 2016 Aug 17;17:646. doi: 10.1186/s12864-016-2984-8 (PMC4987992; doi:10.1186/s12864-016-2984-8)

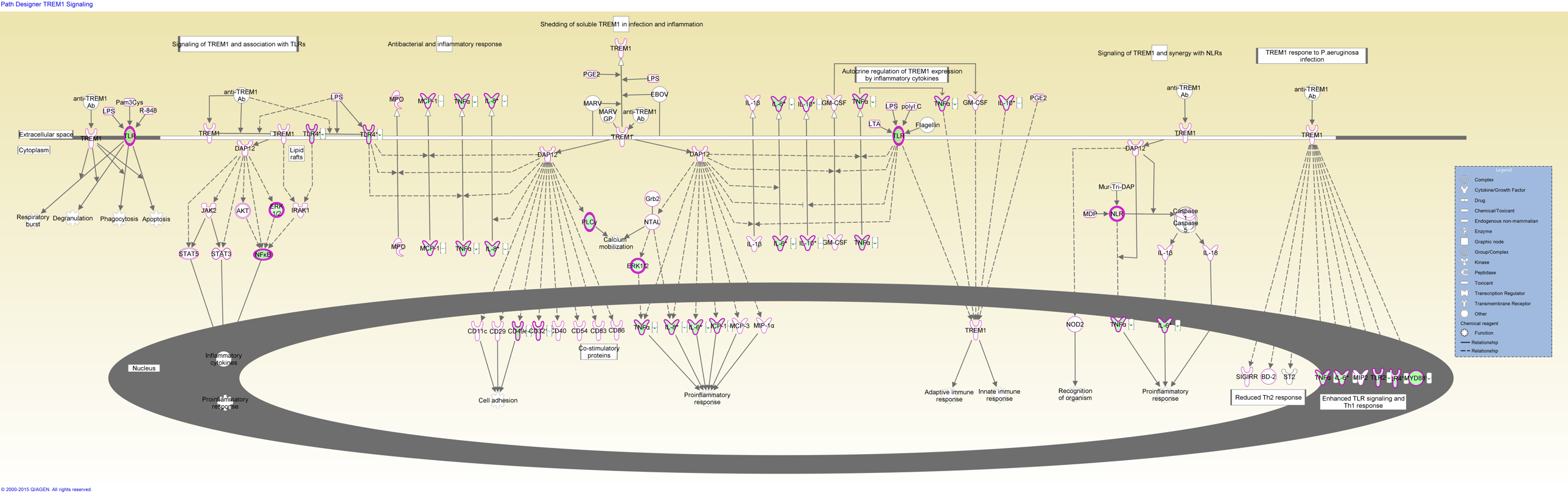

Supplement: Additional file 1: Figure S1. — Alteration of Toll-Like Receptors (TLRs) and the activation of the inflammatory cytokine in parallel with the alteration of the expression level for the adhesion molecules as response to ZEA exposure; pathway generated using IPA (Ingenuity Pathway Analysis). (JPG 5492 kb) [file 12864_2016_2984_MOESM1_ESM.jpg]

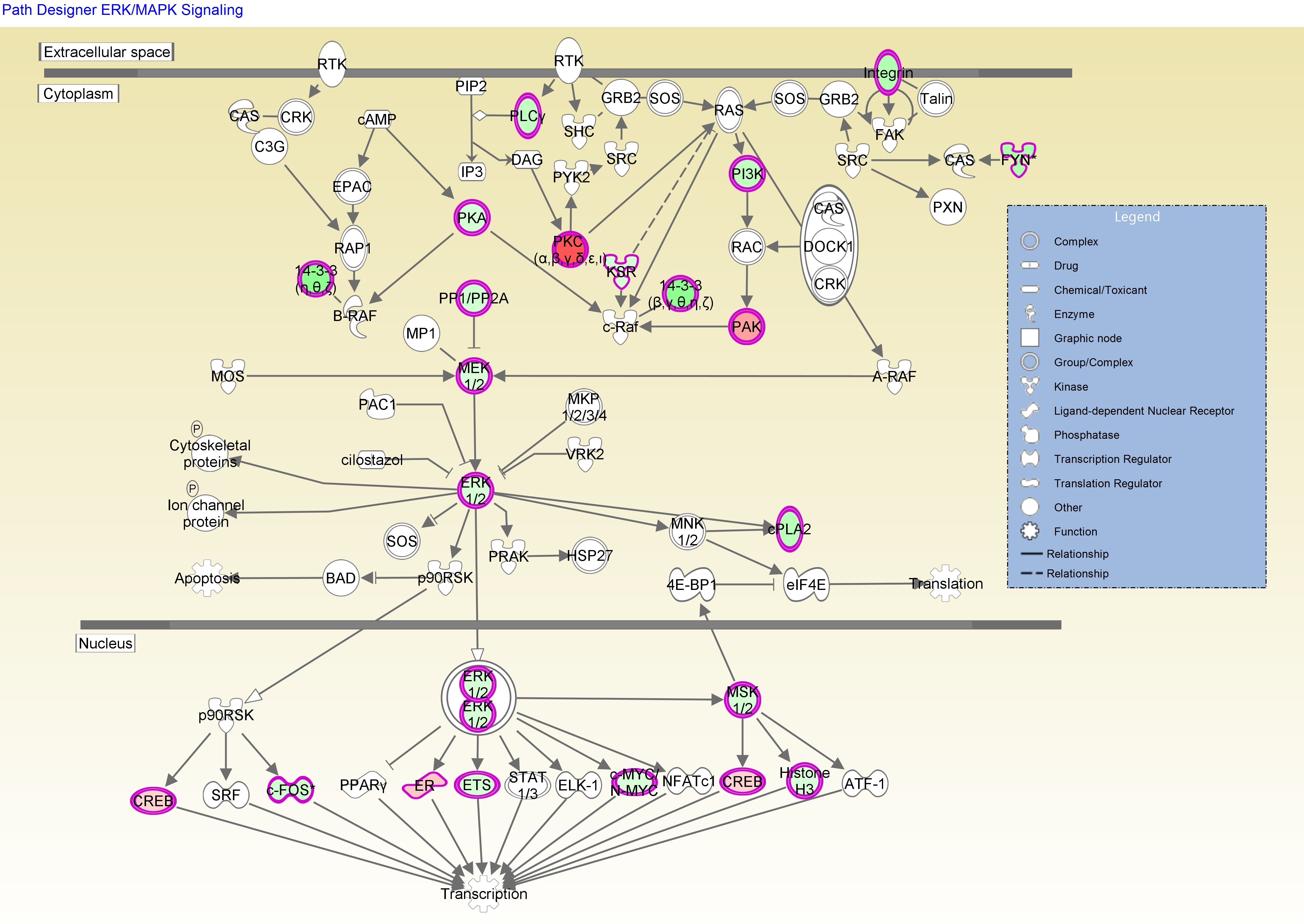

Supplement: Additional file 2: Figure S2. — Alteration of genes related to MAPK (mitogen activate protein kinases), an early event of carcinogenesis, fact demonstrated as response to ZEA exposure; pathway generated using IPA. (JPG 2327 kb) [file 12864_2016_2984_MOESM2_ESM.jpg]

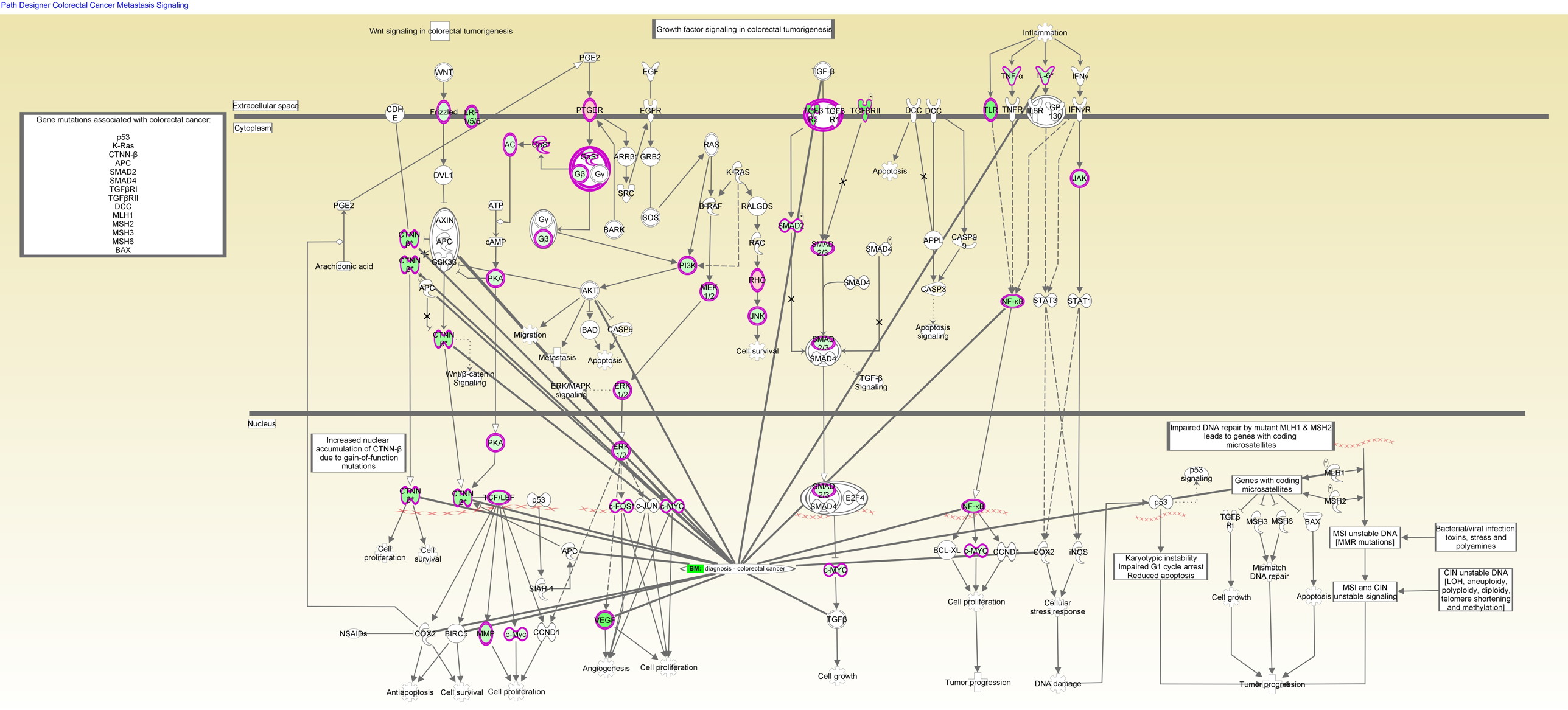

Supplement: Additional file 3: Figure S3. — Activation of colorectal carcinogenic mechanism as response to ZEA exposure; pathway generated using IPA. (JPG 5802 kb) [file 12864_2016_2984_MOESM3_ESM.jpg]

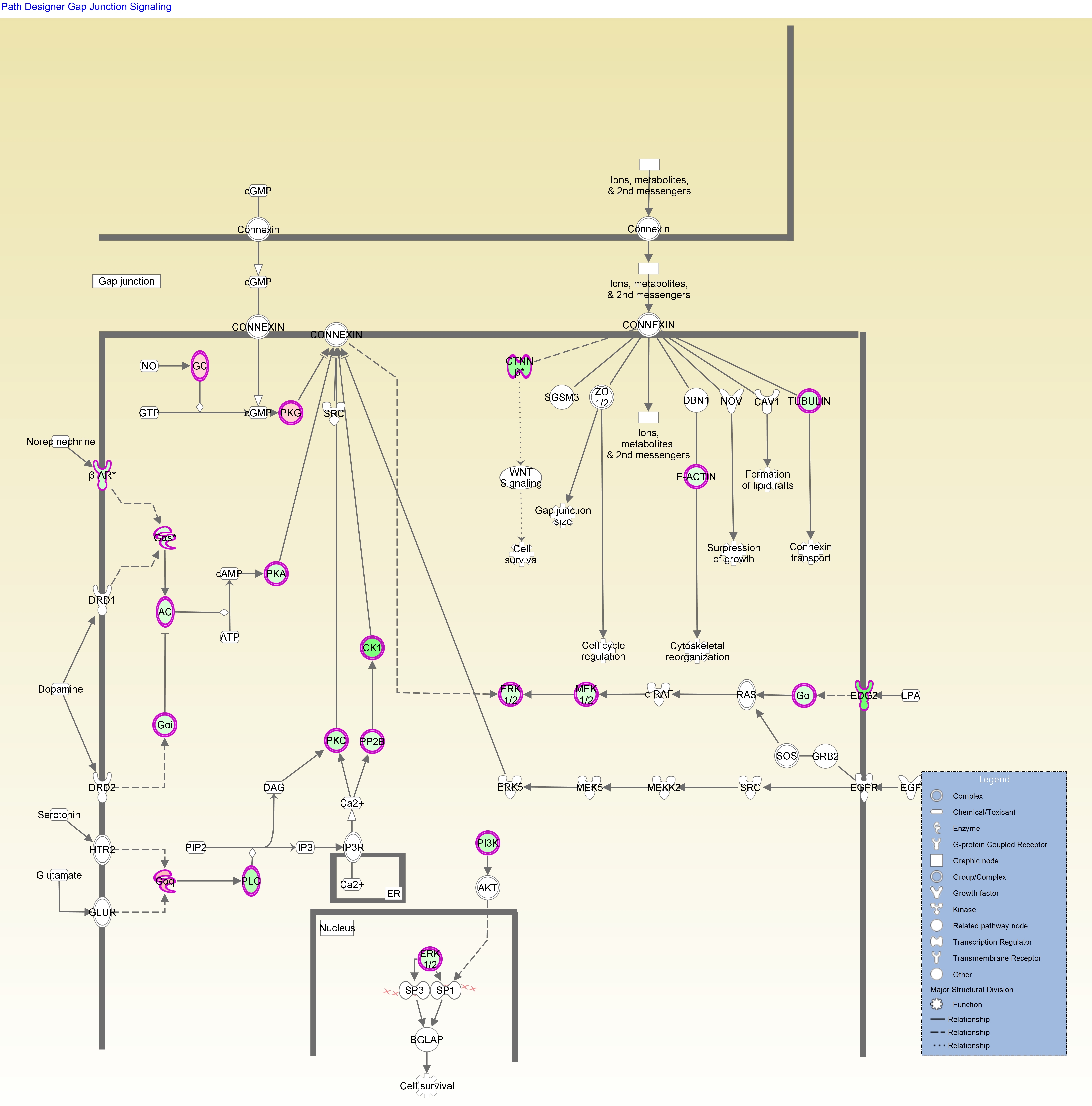

Supplement: Additional file 4: Figure S4. — Alterations in GAP junction signaling with signification on the increasing the susceptibility to bacterial infection; pathway generated using IPA. (JPG 2711 kb) [file 12864_2016_2984_MOESM4_ESM.jpg]
